# Successful In Vitro Shoot Multiplication of *Quercus robur* L. Trees Aged up to 800 Years

**DOI:** 10.3390/plants12122230

**Published:** 2023-06-06

**Authors:** Paweł Chmielarz, Szymon Kotlarski, Ewa Marzena Kalemba, João Paulo Rodrigues Martins, Marcin Michalak

**Affiliations:** 1 Institute of Dendrology Polish Academy of Sciences, Parkowa 5, 62-035 Kórnik, Poland; szymko2@o2.pl (S.K.); jprmartinss@yahoo.com.br (J.P.R.M.); m.michalak@uwm.edu.pl (M.M.); 2 PPHU ASKIK Co., Ltd., Bukowy Las 20, 63-014 Murzynowo Kościelne, Poland; 3 Department of Plant Physiology, Genetics and Biotechnology, University of Warmia and Mazury in Olsztyn, M. Oczapowskiego 1A, 10-721 Olsztyn, Poland

**Keywords:** agar medium, bioreactor system, micropropagation, pedunculate oak, oaks, old trees, RITA^®^

## Abstract

The conservation of the genetic resources of old trees is crucial to their ecological role but is extremely difficult, especially for oak species (*Quercus* spp.) displaying recalcitrance in seed and vegetative propagation methods. Our study aimed to assess the regenerative potential of *Quercus robur* trees of different ages (up to 800 years) during micropropagation. We also aimed to determine how in vitro conditions can influence in vitro regeneration responses. Lignified branches collected from 67 selected trees were cultivated ex vitro in culture pots at 25 °C to obtain epicormic shoots (explant sources). The explants were cultivated on an agar medium supplemented with 0.8 mg L^−1^ 6-benzylaminopurine (BAP) for at least 21 months. In a second experiment, two different shoot multiplication conditions (temporary immersion—RITA^®^ bioreactor and agar medium) and two culture medium formulations (Woody Plant Medium and modified Quoirin and Lepoivre medium) were tested. The results showed that the mean length of the epicormic shoots obtained in a pot culture was a function of donor age and was similar among the group of younger trees (ca. 20–200 years), and varied between older trees (ca. 300–800 years). The efficiency of in vitro shoot multiplication strictly depended on the genotype. A sustainable in vitro culture (defined as survival after 6 months) was only possible for half of the tested old donor trees, even when they survived the first month of in vitro growth. A continuous monthly increase in the number of in vitro cultured shoots was reported in younger oaks and in some old oaks. We found a significant effect of the culture system and the macro- and micronutrient composition on in vitro shoot growth. This is the first report demonstrating that the in vitro culture can be successfully applied to the propagation of even 800-year-old pedunculate oak trees.

## 1. Introduction

The protection of monumental trees, which play an important role in natural ecosystems, is a form of biodiversity protection that enables maintaining the richness and diversity of living organisms from all ecosystems and ecological complexes, together with the diversity of the physical conditions of the habitats in which they occur [1]. The most commonly used method of ex situ conservation of genetic resources is the storage of seeds in gene banks, which allows the long-term storage of genetic resources of plant species, mainly in the form of seeds that tolerate desiccation (orthodox category) [2]. Conversely, the storage of desiccation-sensitive seeds (recalcitrant category), such as oak acorns, remains challenging [3]. When it is not possible to store seeds, the in vitro method allows for the preservation of endangered or valuable plant material via plant tissue cultures of valuable individuals, in addition to their regeneration and reintroduction to the natural environment [4,5,6]. For agricultural species, artificial seeds are produced by encapsulating somatic embryos, shoot tips, or any other micropropagule which has the ability to convert into a plant in vitro or ex vitro [7].

Old trees are a valuable component of forest ecosystems, savannahs, and even urbanized areas [8]. Old forest communities create a suitable microclimate for many species and are a source of food for many animals. They also play an important role by sequestering large amounts of carbon dioxide, which has a direct impact on mitigating the effects of climate change [9]. Old trees display many features valuable for silviculture, and have a cultural and historical value for society; however, in many regions of the world, a rapid decline in the largest and oldest trees has been observed [8]. Extremely old trees living over 1000 years are rare [10]. Among deciduous trees, there are some monumental pedunculate oaks (*Quercus robur* L.) in Europe that are thought to be approximately 1000 years old, such as the Granit Oak in Bulgaria, the Kongeegen in Denmark, the Stelmužė Oak in Lithuania, and the Major Oak in England. In Poland, pedunculate oaks are the oldest tree group, and their ages range from 500–800 years [11].

*Q. robur* is a naturally occurring broadleaved tree in Europe and southeast Asia, where it forms temperate deciduous mixed forests. *Q. robur* grows well in acid soils, although it can grow in soils with higher pH than *Q. petarea.* Fruiting is observed after approximately 40–50 years of growth [12]. The natural regeneration of *Q. robur* is considered to be unsuccessful [13,14]. The phenomenon of dying oak stands has been observed in Poland and Europe since the nineteenth century. Drought, frost damage and defoliation, the long-term impact of key stress factors (climate, habitat), insect gradation, lowered groundwater level, changes in soil chemistry, low temperatures, and environmental pollution are the main causes of the mass death of oak stands [15].

The generative propagation of oaks by seeds is commonly undertaken in afforestation. Traditional techniques of vegetative propagation are of limited use in the case of *Q. robur* because of the low oak shoot rooting rates [16]. The loss of rooting capacity is reported predominantly in adult material, indicating that the ontogenetic state of oak shoots is crucial in in vitro cultures [17]. Micropropagation is one of the in vitro methods of the vegetative multiplication of plant material [18]. Successful micropropagation of *Q. robur* shoot sections with the apical meristem of shoots [19,20] or plumules [21] is possible when placed on a certain medium and cultured under specific temperature and lighting conditions [22]. However, the selection of the best explant source determines the success of micropropagation [23]. An important factor in plant tissue culture is the medium. Both Woody Plant Medium (WPM) and Quoirin and Lepoivre (QL) [24] media can be successfully used in oak micropropagation [25,26]. Increased multiplication efficiency can also be achieved by the cultivation in bioreactors using a temporary immersion bioreactor (TIB) or temporary immersion systems (TIS) [27]. The TIB system was found to be very efficient for the in vitro mass production of genetically homogeneous horticulture and medicinal species [28]. *Q. robur* shoots were successfully micropropagated in liquid culture in a Plantform^TM^ bioreactor [29]. Recently, morphophysiological disorders that may occur during in vitro regeneration of juvenile *Q. robur* explants on medium with cytokinins were investigated in order to establish an optimized micropropagation protocol [30]. The acclimation to ex vitro conditions is an important stage of micropropagation. Ectomycorrhiza inoculation can improve the water and physiological condition of plants during the adaptation phase [31].

The success of the micropropagation of woody plants is closely related to their age [32]. The initiation of tissue cultures from old trees is hampered by the high percentage of microbial contamination and higher sensitivity to disinfectants [33]. Therefore, in our study, we aimed to test the hypothesis that the ability of oak trees to undergo in vitro micropropagation decreases with age. The possibility of in vitro cultivation of plant material from individuals aged 70–300 years has been previously reported [20]. However, to the best of our knowledge, there are no studies demonstrating the possibility of cloning oaks older than 500 years. We hypothesized that the micropropagation of an approximately 800-year-old *Quercus robur* L. is possible. Our study thus aimed to assess the regenerative potential of *Q. robur* trees of different ages (ca. 20–800 years) during micropropagation. Furthermore, we aimed to determine how in vitro conditions (agar medium formulation and TIS) influence in vitro regeneration responses.

## 2. Results

### 2.1. Ex Vitro Cultivation of Branches in Pots

**Figure 1:**
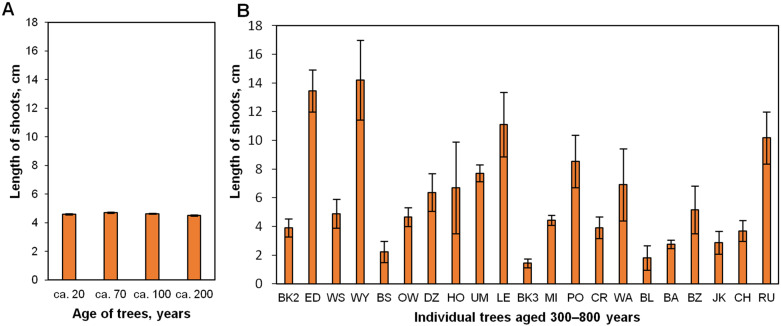
The average length of epicormic shoots (per a lignified shoot) obtained after four weeks of pot culture of *Quercus robur* plant material. Lignified branches were collected from two groups of mature trees. (**A**) Forty-six trees pooled in four categories, aged ca. 20–200 years, (**B**) Twenty-one monumental trees aged ca. 300–800 years (±50). Data are the means of three biological replicates ± standard error. BK2—Bąkowski 2 Oak (ca. 300 years old); ED—Edward Oak (ca. 350); WS—Władysława Szafera Oak (ca. 400); WY—Wybickiego Oak (ca. 400); BS—Bolesław Oak (ca. 400); OW—Owińska Oak (ca. 500); DZ—Dziadziuś Oak (ca. 500); HO—Hoggo Oak (ca. 500); UM—Uparty Mazur Oak (ca. 500); LE—Lech Oak (ca. 550); BK3—Bąkowski 3 Oak (ca. 550); MI—Mieszko I Oak (ca. 650); PO—Poganin Oak (ca. 650); CR—Chrześcijanin (ca. 650); WA—Warcisław Oak (ca. 650); BL—Bolko Oak (ca. 650); BA—Bartek Oak (ca. 700); BZ—Bażyńskiego Oak (ca. 700); JK—Jan Kazimierz Oak (ca. 700); CH—Chrobry Oak (ca. 800), RU—Rus Oak (ca. 800).

Significant differences were observed in the average length of the epicormic shoots obtained from trees of different ages (Figure 1). The length of epicormic shoots obtained from trees aged ca. 20–200 did not differ significantly (4.51–4.7 cm) (Figure 1A), whereas the length of shoots was more diversified in older trees (Figure 1B). The analysis of variance components (REML method) revealed that, as a component, the monumental tree genotype carries 85.3% of the variation in the average number of shoots produced by the tree (Table S1).

**Figure 2:**
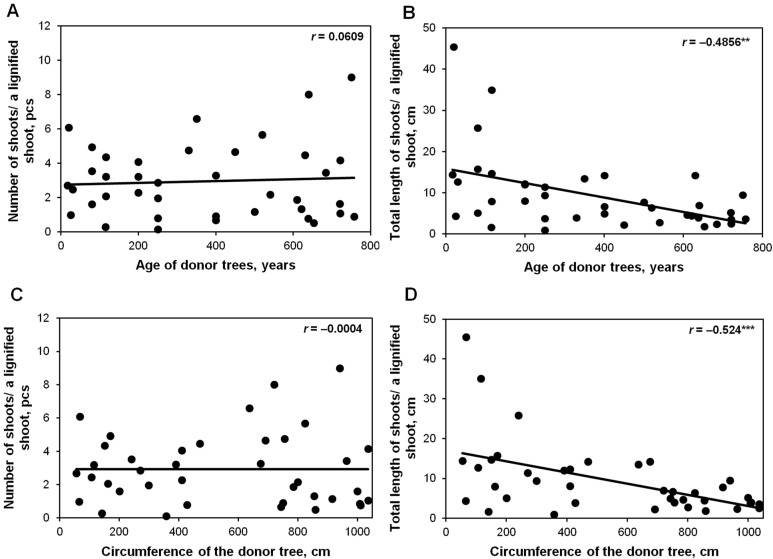
Pearson correlation coefficient (*r*) between donor tree age and average total length (**A**) and the number (pcs-pieces) of epicormic shoots obtained as a result of pot culture (**B**) and between donor tree circumference and average number (**C**) and the average total length (**D**) of epicormic shoots. The correlation is significant at the ** *p* ≤ 0.01 or *** *p* ≤ 0.001 level.

No significant correlations were found between the donor tree age and the average number of epicormic shoots obtained from ca. 20–200-year-old trees (Figure 2A). The effect of donor tree age was confirmed by an average negative correlation between mean total shoot length and donor tree age (*r* = −0.4856, *p* ≤ 0.01) (Figure 2B). There was also no significant correlation between the mean number of shoots and donor tree circumference (Figure 2C), whereas a high negative correlation (*r* = −0.524, *p* ≤ 0.001) was found between mean total shoot length and donor tree circumference (Figure 2D).

### 2.2. Effect of Donor Tree Age on Explant Survival after the First Month of In Vitro Culture

**Figure 3:**
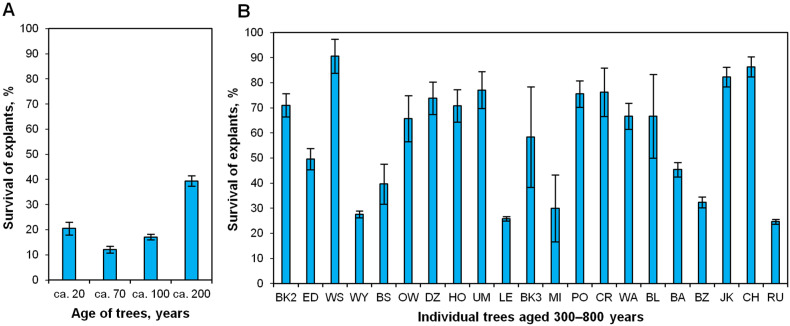
Explant survival during the first month of in vitro culture corresponding to two groups of *Q. robur* mature trees. (**A**) Forty-six trees pooled in four categories, aged ca. 20–200 years, (**B**) Twenty-one monumental trees aged ca. 300–800 years (±50). Data are the means of three biological replicates ± standard error. BK2—Bąkowski 2 Oak (ca. 300 years old); ED—Edward Oak (ca. 350); WS—Władysława Szafera Oak (ca. 400); WY—Wybickiego Oak (ca. 400); BS—Bolesław Oak (ca. 400); OW—Owińska Oak (ca. 500); DZ—Dziadziuś Oak (ca. 500); HO—Hoggo Oak (ca. 500); UM—Uparty Mazur Oak (ca. 500); LE—Lech Oak (ca. 550); BK3—Bąkowski 3 Oak (ca. 550); MI—Mieszko I Oak (ca. 650); PO—Poganin Oak (ca. 650); CR—Chrześcijanin (ca. 650); WA—Warcisław Oak (ca. 650); BL—Bolko Oak (ca. 650); BA—Bartek Oak (ca. 700); BZ—Bażyńskiego Oak (ca. 700); JK—Jan Kazimierz Oak (ca. 700); CH—Chrobry Oak (ca. 800), RU—Rus Oak (ca. 800).

Survival of explants originating from ca. 20–200-year-old oaks after the first month of in vitro culture was 12–40% (Figure 3A). Explants derived from 21 monumental oaks aged ca. 300–800 years were characterized by more diverse survival rates in comparison to younger oaks (Figure 3B). The highest survival rate for explants from older oaks was 90%, and the lowest was approximately 23% (Figure 3B).

### 2.3. Effect of a Donor Tree and the Number of Subcultures (up to 21 Months) on In Vitro Shoot Proliferation

**Figure 4:**
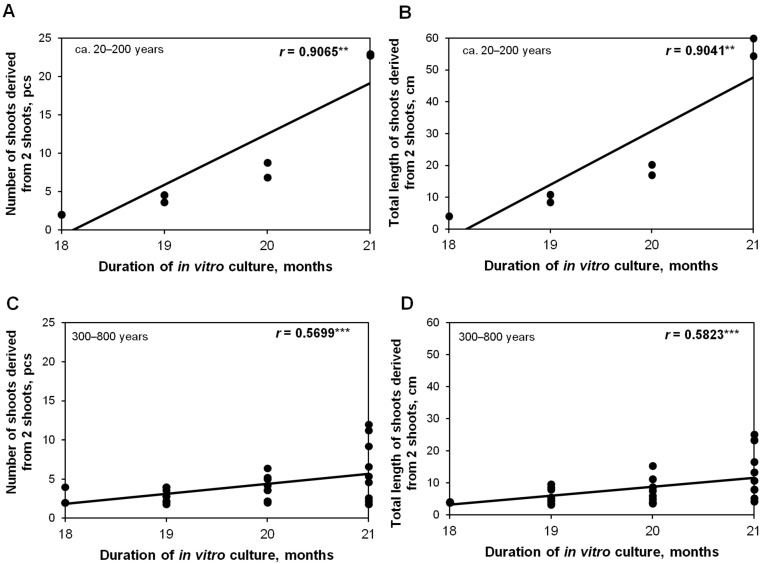
Pearson correlation coefficient (r) between the number (pcs-pieces) (**A**,**C**) or total length of shoots (**B**,**D**) and in vitro culture duration from 18 to 21 months in oaks aged ca. 20–200 years (**A**,**B**) and monumental oaks (ca. 300–800 years old) (**C**,**D**). Correlation significant at the ** *p* ≤ 0.01 or *** *p* ≤ 0.001 level. The x-axis represents the months from the start of the culture after sterilization.

**Figure 5:**
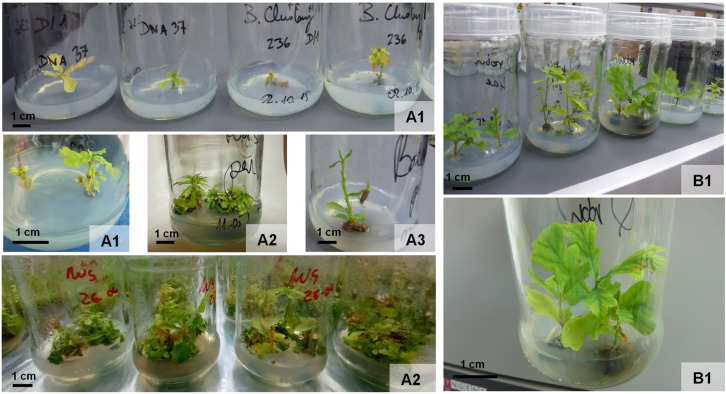
Shoots derived from monumental oaks (ca. 300–800 years: Chrobry Oak (ca. 800) (**A1**), Rus Oak (ca. 800 (**A2**), Bolesław Oak (ca. 400) (**A3**), and younger oaks, ca. 20–200 years (**B1**) after four weeks of proliferation.

The shoot number and the total length of new shoots (starting from two initial shoots for each repetition) for different genotypes were recorded monthly during four successive subcultures up to 21 months. The genotype, the number of subcultures, and their interaction had a significant effect on the proliferation of micropropagated oaks (Table S2). In the case of oaks aged ca. 20–200 years, positive correlations were reported between the subculture number and the number of shoots (r = 0.9065, *p* ≤ 0.001; Figure 4A) and with the shoot length (r = 0.9041, *p* ≤ 0.001, Figure 4B). This demonstrates a continuous monthly increase in the number of shoots cultured in vitro during the studied four-month period. For the oldest oaks, both correlations were weaker in comparison to the youngest oaks; in addition, a sustainable culture (shoots growing in vitro > 6 months) was only possible for half of the tested donor trees (ca. 300–800 years old). A positive correlation was found between the number of shoots multiplied during a 4-month in vitro culture from monumental oaks and the duration of this culture (*r* = 0.5699, *p* ≤ 0.001) (Figure 4C). A positive correlation was also found between the total shoot length (*r* = 0.5823, *p* ≤ 0.001) and culture duration (Figure 4D). Representative images of shoots derived from monumental oaks after four weeks of proliferation are provided in Figure 5.

### 2.4. Efficiency of Shoot Multiplication in the RITA^®^ Bioreactor

**Figure 6:**
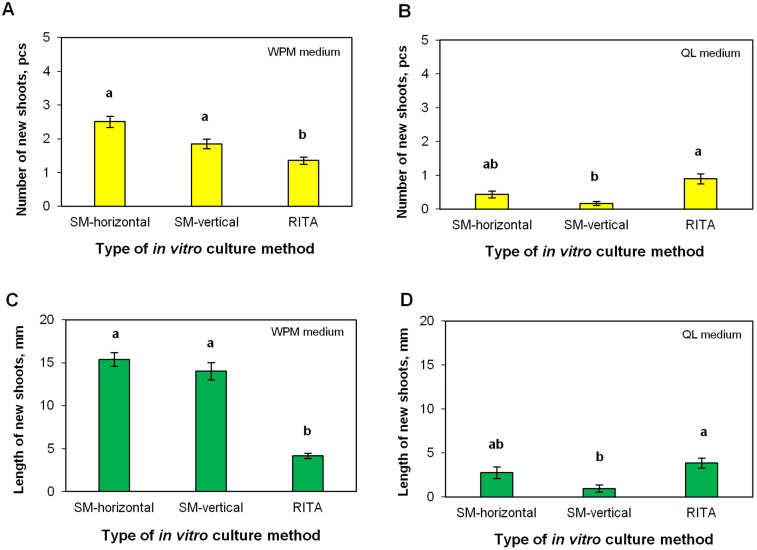
Comparison of the number of new shoots (**A**,**B**) and average length of new shoots (**C**,**D**) after one month of in vitro culture of genotype Bąkowski 2 Oak on solid medium (SM) or in the RITA^®^ bioreactor using WPM (**A**,**C**) or QL medium (**B**,**D**) and horizontal (SM-horizontal) or vertical (SM-vertical) explant arrangements. Data are the means of three biological replicates ± standard error. Different letters indicate significant differences according to Tukey’s post hoc test.

This experiment tested the multiplication of shoots in in vitro culture on two agar media (WPM, QL), where the shoots were arranged horizontally or vertically, and on liquid media (WPM, QL) in the RITA bioreactor. The WPM and QL agar media differed in the efficiency of shoot multiplication. The number of shoots was higher when shoots were cultured on WPM (1.36–2.51) than when shoots were cultured on QL medium (0.16–1.08). The shoots were also longer when cultured in WPM (4.17–15.39 mm) than in QL medium (0.96–3.86 mm) (Figure 6). The analysis of variance revealed a statistically significant effect of the medium on shoot growth (Table S3). The average number and length of new shoots grown on agar medium did not differ according to the horizontal or vertical arrangement of explants (Figure 6). Importantly, significantly fewer and shorter new shoots were obtained when their propagation was carried out in the RITA^®^ bioreactor (best variant: 1.36 pcs, 4.17 mm average length) compared to the culture on agar WPM (best variant: 2.17 pcs, 14.71 mm average length). Both shoot necrosis and any other abnormalities in growth were not observed. Cultivation in the RITA^®^ bioreactor with the QL medium allowed us to obtain significantly more (0.90 pcs) and longer (3.86 mm) new shoots than in the culture on agar medium when the shoots were placed vertically in it (0.16 pcs, 0.96 mm) (Figure 6).

## 3. Discussion

### 3.1. Epicormic Shoot Formation in Pot Culture

Clonal propagation of the selected plant species that are recalcitrant to conventional propagation methods is challenging [34]. Following our previous studies on monumental oaks [35,36], we can here confirm our hypothesis and present the first evidence of an effective propagation method for *Q. robur* trees aged up to 800 years. The pot culture of lignified shoots of *Q. robur* enabled us to obtain epicormic shoots from all 21 tested monumental oaks that served as explants for the initiation of in vitro culture. The possibility of obtaining epicormic shoots for *Q. robur* is crucial in the initiation of in vitro cultures. Old plants have a lower ability to initiate in vitro culture, and for many species, the method is not very effective [20,37,38]. Recently, miRNAs involved in epigenetics were suggested as key regulators of vegetative phase change and exogenously induced plant rejuvenation and regrowth [39]. The rapid growth and high regenerative capacity of epicormic shoots depended on the time of harvest of the plant material. In our study, the collection of branches in April (before winter bud burst) resulted in the successful growth of epicormic shoots developed from dormant buds. The optimal time of harvest of plant material depends on the species, and can occur from mid-winter to late spring [38]. Two North American oak species, *Q. alba* and *Q. rubra*, displayed the best epicormic bud sprouting success when lignified shoots were collected from February to April [40]. To the best of our knowledge, *Eucalyptus globulus*, growing in a temperate environment, was the only woody plant reporting no seasonal effects on explant shoot production [41]. Another factor determining the success of explant growth in our study was to use only larger branches with epicormic buds, while thinner branches were cut off together with winter buds. This was also reported for other species, such as *Robinia pseudoacacia* [42]. In our study, tree circumference was also responsible for the success of epicormic shoot growth. We showed an inverse correlation between the average total length of epicormic shoots and both the age (ca. 20–800 years) and circumference (0.15–10.4 m) of the donor tree. These results are in agreement with previous observations [37,40,41].

### 3.2. Donor Tree Age, Genotype, and In Vitro Shoot Regeneration

According to many reports, the age of the donor tree has a decisive influence on the shoot regeneration capacity in tissue cultures [43,44]. We can confirm our hypothesis that in vitro shoot multiplication of older oaks is possible. After the first month of in vitro culture, some tree material was characterized by a high survival and growth rate, while others exhibited a low survival rate and a tendency to die. San-José et al. [45] observed the better response of de novo shoot formation when explants were re-cultured on the same medium, and later there was a decay in shoot production. The stabilization of in vitro cultures, measured in our experiment as the number of multiplied shoots from individual trees, occurred about six months after the initiation of a culture, depending on the tree genotype. The regeneration capacity could be linked to a hormonal balance (the content and biosynthesis of plant hormones) and/or epigenetics [44,46]. According to the cited studies, the endogenous auxin/cytokinin levels may decrease, and the DNA methylation levels may increase as the donor tree ages. Therefore, we hypothesized that the explants obtained from monumental trees with lower regenerative capacity could be the result of a higher level of methylation and the lower biosynthesis of hormones. During in vitro culture, organogenic responses are affected by the interaction between plant growth regulators added to the culture medium and the endogenous hormone content. Exogenous cytokinins are essential to shoot formation during the in vitro culture of oaks, but the use of synthetic cytokinins, such as 6-benzylaminopurine (BAP), may show a residual long-term effect, interfering with later subcultures [47,48]. The lack of a clear pattern in the results encouraged us to prolong the culture time and assess the effect of culture duration (up to 21 months) on the efficiency of shoot multiplication expressed as the number and total length of the multiplied shoots. We observed, during the 21-month shoot culturing period, a continuous monthly increase in the number of in vitro cultured shoots in younger oaks and for some old oaks. The efficiency of in vitro culture of shoots from both younger oaks (ca. 20–200 years old) and monumental oaks (ca. 300–800 years old) was correlated with the duration of culture, and was evident during the initiation of in vitro cultures. In this study we used a large number (67) of mature trees that showed a high variability in regenerative capacity, ranging from successful proliferation for more than 20 months to a sharp decay during the first months, which prevented further propagation. Similarly, Juncker and Favre [49], in a study of 150-year-old *Q. robur* trees, found that the genotype has a large impact on the ability to micropropagate because some individuals died in the initial period of the culture, whereas others showed a gradual decrease in vitality, and most juveniles showed increasing growth vigor over time.

### 3.3. Multiplication of Shoots in the RITA^®^ Bioreactor

Numerous scientific reports indicate the possibility of increasing the efficiency of plant material propagation using bioreactors operating with a temporary immersion system [50,51,52], including the RITA^®^ bioreactor [53,54]. The advantages of this system are the short-term contact of explants with the liquid medium, the easy availability of micro- and macronutrients, efficient gas exchange [50], and large-scale multiplication [52]. An increase in shoot multiplication efficiency in the bioreactor was obtained for other trees, such as eucalyptus [53], apple [55], chestnut [56] and willow [57]. To the best of our knowledge, this is the first attempt at culturing axillary shoots of adult oak trees in bioreactors. In the present study, the efficiency of oak shoot multiplication in the RITA^®^ bioreactor was lower than on an agar medium. We observed a lower average number (1.4 pcs) and length (4.2 mm) of new shoots obtained with the former, whereas the proliferation was higher with the conventional culture on agar media (an average of 1.9–2.5 pcs and 14.0–15.4 mm in length). The reports on the use of bioreactors for oak proliferation are scarce. Regarding axillary shoots, there is a report on the use of the Plantform™ bioreactor for the micropropagation of *Q. robur* shoots originating from seedlings, which did not cause a significant increase in the number of new shoots obtained compared to standard cultivation on a solid agar medium [29]. These authors used a different immersion/aeration regime and reported hyperhydricity when the explants were submitted to a longer immersion period. Although the proliferation of their juvenile material was higher than that observed with old oaks in this study, it should be improved in terms of being an alternative to agar-based media. In the case of somatic embryos, multiplication rates and somatic embryo quality in two embryogenic lines of *Quercus robur* derived from mature trees were investigated by Mallón et al. [58], who tested several immersion cycles. In this case, higher proliferation was obtained by TIS, which also had a significant effect on somatic embryo synchronization, as it enabled a higher production of cotyledonary embryos. The success of cultivation in a batch flood bioreactor depends on many factors, such as the container size, the ratio of medium volume per explant, and flooding cycle length and frequency [59]. In the case of *Q. robur* axillary shoots, further research using different times and frequencies of flooding cycles, as well as different volumes of medium and amounts of explants, is needed to increase the efficiency of multiplication in a bioreactor in the future. 

The composition of the medium has a significant impact on the efficiency of in vitro plant multiplication. Both on the agar medium and on the TIS, a lower number of new shoots was observed on the modified QL medium as compared to the WPM in this study. The WPM medium is widely used during the in vitro multiplication phase for woody species during the in vitro multiplication phase of the genus *Quercus* [60,61]. Compared to QL, the WPM formulation has a higher nitrogen content, an essential macronutrient, and a structural component in plants. Furthermore, it is important to point out that nitrate (NO_3_) can stimulate a transient increase in endogenous cytokinin levels [62]. Martins et al. [30] also found that the addition of available nitrogen in the culture medium potentiated the effect of BAP on in vitro multiplication. Contrary to this, Wesoły et al. [63] obtained good results on the multiplication of the seedlings of pedunculate oak using a modified QL medium.

### 3.4. Shoot Positions on Agar Media

Species of the genus *Quercus* are recalcitrant, i.e., recalcitrant in terms of seed physiology as well as micropropagation [64]. Additionally, episodic growth, stunting, or the lethality of multiplicated *Quercus* shoots is reported when the explants are vertically positioned in the culture [65]. However, the shoot multiplication efficiency of in vitro cultured explants originating from *Q. robur* trees was comparable when the shoots were placed on the medium in a vertical or horizontal position, similar to the study of San-José et al. [45], on plant material from 15- and 75-year-old *Q. robur* trees. Interestingly, significantly more shoots were obtained when explants from *Q. rubra* shoots aged 3 months, 4 years, and 30–40 years [66], as well as from 100-year-old *Q. robur* trees [20] were placed horizontally on the medium. These divergent results support the need for further research to continuously improve in vitro culture protocols, because the distribution and transport of endogenous growth regulators and the availability of nutrients might depend on the initial position of shoots in the medium. The different responses of horizontally positioned shoots were related to the donor tree age.

## 4. Materials and Methods

### 4.1. Plant Material

Plant material was collected from 67 mature *Quercus robur* trees growing in Poland. Forty-six of them were ca. 20–200 years old, and twenty-one were monumental oaks with ages ranging from ca. 300 to 800 years [35]. Lignified branches of 1.5 m in length were collected in late April of 2014. Branches with a diameter of approximately 2–4 cm were cut into 30–40 cm sections (thin branches with winter buds were removed) and transported to the laboratory within 24 h.

### 4.2. Initiation of Epicormic Shoots in Pot Culture (Ex Vitro)

**Figure 7:**
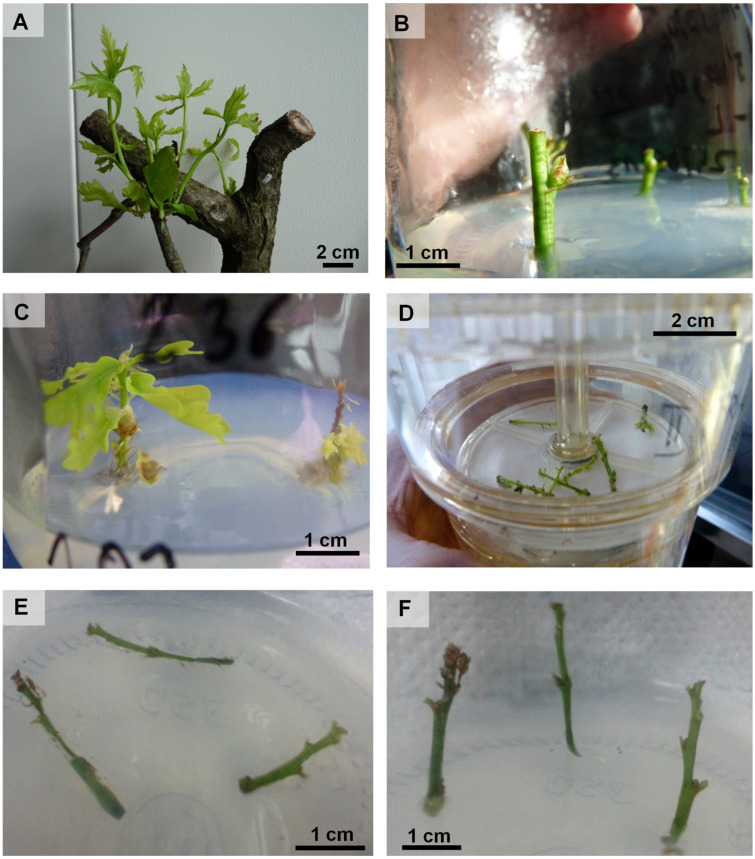
Initiation of *Quercus robur* in vitro culture: epicormic shoots growing from branches (**A**), an explant (from epicormic shoots) with a bud (**B**). Multiplication of shoots on agar medium (**C**); in the bioreactor RITA^®^ (**D**); and on agar medium positioned horizontally (**E**) or vertically (**F**).

Lignified branches (lignified shoots) of ca. 40 cm in height with a diameter of 1–4 cm with epicormic buds located under the bark were cultivated in water in 500-mL buckets at a temperature of 25 °C. To ensure good water conduction inside the lignified branches, the conducting bundles were vented by placing the lower ends of the branches in boiling water for 2–5 min. The shoots were then surface-decontaminated by immersion in a 10% sodium hypochlorite solution for 10 min. The branches were placed in a growth room with a light intensity of 40 µmol m^−2^ s^−1^ photosynthetically active radiation (PAR), a 16 h light/8 h dark photoperiod, and an air humidity of 80–90%. Epicormic shoots were developed from dormant buds located under the bark (Figure 7A). To evaluate the epicormic shoot regeneration, the number and total length (in cm) of all epicormic shoots growing from one lignified shoot were recorded. The number of epicormic shoots and their total length were a mean of three replicates (five lignified shoots in each) of one tree.

### 4.3. Establishment of In Vitro Culture from Explants and Further Shoot Multiplication

The explants (Figure 7B) were disinfected and introduced to in vitro conditions as proposed by Kotlarski et al. [35] and Martins et al. [30]. The explants were cultured in an agar (7 g L^−1^) woody plant medium (WPM) [67] supplemented with 0.8 mg L^−1^ BAP and 30 g L^−1^ sucrose. The BAP concentration was chosen in accordance with Puddephatt et al. [68]. The cultivation was carried out under controlled conditions in a phytotron chamber under a light intensity of 77 µmol m^−2^s^−1^, over a 16 h light/8 h dark photoperiod, and at 20 °C. The in vitro plant material (Figure 7C) was transferred to fresh medium with the same formulation described above every four weeks and cultivated for at least 21 months. After one month of in vitro culture, the survival rate of shoots from younger oaks (46 trees pooled in four categories: ca. 20, ca. 70, ca. 100 and ca. 200 years old) and old oaks (21 trees, ca. 300–800 years old) were measured. In the experiment, five repetitions (two shoots in one jar in each repetition) per tree were used, and the initial length of the shoots was 2–3 cm.

### 4.4. In Vitro Multiplication of Shoots Originating from Trees of Different Ages

The regeneration capacity during the in vitro multiplication of *Q. robur* shoots (Figure 7C) was tested. After 18 months of continuous subcultures, the plant material was divided into two groups. One group consisted of 46 trees pooled in four categories aged ca. 20–200 years, and the second group of 21 trees was aged ca. 300–800 years. These genotypes were cultured for four months in the conditions described above. Every four weeks the shoots were transferred to fresh media and the shoot number and length were recorded. For each genotype, five replicates of two shoots each were used, and the initial length of the shoots was 2–3 cm.

### 4.5. In Vitro Multiplication in the RITA^®^ Bioreactor System and Agar Medium

For this experiment we selected the Bakowski 2 Oak genotype (aged ca. 300 years) due to its good regenerative capacity over 10 months of culturing. The experiment was performed to test several factors: (i) two in vitro conditions: a temporary immersion system with RITA^®^ bioreactors (Figure 7D), and the conventional agar medium, (ii) two media formulations, WPM and QL, the composition of which can be found in Table S4, and (iii) the explant arrangement in the medium with agar: horizontal (Figure 7E) or vertical (Figure 7F). All media were supplemented with 0.8 mg L^−1^ of BAP. In the RITA^®^ bioreactor, the explants were immersed for 4 min every 3 h. Initial explants were 2 cm shoots without leaves. The number and length of new shoots were recorded after four weeks. The experiment design was completely randomized, and samples consisted of three replicates per treatment, and each replicate consisted of 15 explants. 

### 4.6. Statistical Analysis

The obtained data were subjected to an analysis of variance (ANOVA), and the significance of differences was tested using Tukey’s test at a significance level of *p* ≤ 0.05. A multivariate ANOVA (MANOVA) was used for the data analysis of repeated measures. For percentage data analysis, values were arc-sin-transformed. In justified cases, a restricted maximum likelihood (REML) analysis of the effects of variance components was performed. The correlation coefficient was determined for the selected data using Pearson’s R method. To interpret the correlation strength, the classification of Guilford [69] was adopted. All statistical analyses were performed using SAS JMP^®^ Pro 16.

## 5. Conclusions

In this study, we demonstrated the successful in vitro shoot multiplication of ca. 800-year-old *Q. robur* trees for the first time, thereby confirming our hypothesis. However, some old oak trees appeared to be recalcitrant to micropropagation. The effect of genotype and tree age was significant on the efficiency of epicormic shoot formation during the pot culture (explants preparation) and shoot multiplication in vitro. Generally, younger oaks displayed a higher potential of in vitro growth. The survival (up to one month) of explants during the in vitro culture did not guarantee the maintenance of the culture for longer than periods of six months. The sustainable in vitro culture of the oldest oaks was only possible for some of the 21 tested monumental trees, those exhibiting a multiplying rate similar to younger oaks. It was also found that RITA^®^, the temporary immersion bioreactor system, did not increase the multiplication rate for old oaks in comparison to the agar medium. This study demonstrates that we can protect the genotypes of ca. 800-year-old *Q. robur* trees using an optimized micropropagation protocol.

## Data Availability

The data presented in this study are available in the article and in the Supplementary Material.

## Supplementary Materials

The following supporting information can be downloaded at https://www.mdpi.com/article/10.3390/plants12122230/s1; Table S1: Average number of shoots produced by monumental trees (ANOVA); Table S2: Average number of shoots and their length at the 18th to the 21st month of cultivation (MANOVA); Table S3: The effect of medium on shoot production; Table S4: The composition of agar media.
