# Supplementary material for: Successful In Vitro Shoot Multiplication of Quercus robur L. Trees Aged up to 800 Years
Source: Plants (Basel). 2023 Jun 6;12(12):2230. doi: 10.3390/plants12122230 (PMC10301615; doi:10.3390/plants12122230)
Supplement: Supplementary file 1 [file plants-12-02230-s001.zip › plants-2345976-supplementary.pdf]

SUPPLEMENTAL DATA

Table S1. Analysis of variance components using the residual maximum likelihood (REML) method for the average number of shoots produced by 21 monumental trees. Percentage of variance components (%) was calculated using Wald chi-squared test.

Factor	Variation	Components	Wald test	%
Tree	5.788951	4.430038	***p* = 0.0034 ***	85.27
Others		0.65257		14.73
Sum		5.195295		100

* Significant differences

Table S2A. Analysis of variance (MANOVA) for the average number of shoots produced in in vitro cultures by trees of different ages, in the period from the 18th to the 21st month of cultivation. NumDF, degrees of freedom in the numerator; denDF, DF in the denominator.

	Test	F	NumDF	DenDF	*p*
Tree	F	3.1168	10	44	**0.0044** *
Time	F	15.7938	3	42	**<0.0001** *
Interaction					
Tree x Time	Wilks' Lambda	2.7204 [1]	30	123.95	**<0.0001** *

* Significant differences
[1] Approximated F value

Table S2B. Analysis of variance (MANOVA) for the average length of shoots produced in in vitro cultures by trees of different ages, in the period from the 18th to the 21st month of cultivation. NumDF, degrees of freedom in the numerator; denDF, DF in the denominator.

	Test	F	NumDF	DenDF	*p*
Tree	F	3.6892	10	44	**0.0044** *
Time	F	24.6808	3	42	**<0.0001** *
Interaction					
Tree x Time	Wilks' Lambda	34332 [1]	30	123.95	**<0.0001** *

* Significant differences
[1] Approximated F value

Table S3A. Analysis of variance for the average number of new shoots grown under different conditions on WPM medium. Df, degrees of freedom; SS, sum of squares; MS, mean squares.

	**Df**	**SS**	**MS**	**F**	***p***
Type of culture	2	8988.937	4494.47	63.0215	**<0.0001** *
Error	357	25459.96	71.32		
Total	359	34448.9			

* Significant differences

Table S3B. Analysis of variance for the average number of new shoots grown under different conditions on BG1 medium. Df, degrees of freedom; SS, sum of squares; MS, mean squares.

	**Df**	**SS**	**MS**	**F**	***p***
Type of culture	2	206.0802	103.04	6.9276	**0.0013** *
Error	141	2097.215	14.874		
Total	143	2303.295			

* Significant differences

Table S3C. Analysis of variance for the average number of new shoots grown under different conditions on WPM medium. Df, degrees of freedom; SS, sum of squares; MS, mean squares.

	**Df**	**SS**	**MS**	**F**	***p***
Type of culture	2	79.90556	39.9528	17.2493	**<0.0001** *
Error	357	826.8833	2.3162		
Total	359	906.7889			

* Significant differences

Table S3D. Analysis of variance for the average number of new shoots grown under different conditions on modified QL medium. Df, degrees of freedom; SS, sum of squares; MS, mean squares.

	**Df**	**SS**	**MS**	**F**	***p***
Type of culture	2	13.0417	6.52083	11.9472	**<0.0001** *
Error	141	76.95833	0.5458		
Total	143	90			

* Significant differences

Table S4. The composition of modified Quoirin and Lepoivre (QL) and Woody Plant Medium.

**Macronutrients**	**QL (mg· L^{-1})**	**WPM (mg· L^{-1})**
NH_4NO_3	50.00	400.00
K_2SO_4	800.00	990.00
$CaCl_2$	-	72.50
$Ca(NO_3)_2·4H_2O$	1000.00	471.26
$MgSO_4·7H_2O$	200.00	180.54
KH_2PO_4	200.00	170.00
**Micronutrients**	**QL (mg· L^{-1})**	**WPM (mg· L^{-1})**
FeEDDHA	100.00	36.70
$ZnNa_2EDTA$	10.00	-
$ZnSO_4·7H_2O$	-	8.60
H_3BO_3	8.00	6.20
$MnSO_4·H_2O$	10.00	22.30
$Na_2MoO_4·2H_2O$	0.25	0.25
$CuSO_4·5H_2O$	0.20	0.25
$CoCl_2·6H_2O$	0.02	-
$NiCl_2·6H_2O$	0.01	-
KI	0.20	-
**Vitamins and aminoacids**	**QL (mg· L^{-1})**	**WPM (mg· L^{-1})**
m-inositol	200.00	100.00
thiamine	2.00	1.00
pyridoxine	0.50	0.50
nicotinic acid	2.00	0.50
glycine	2.00	2.00
**pH**	**5.8**	**5.8**
